# Characteristics of Skeletal Muscle Fibers of SOD1 Knockout Mice

**DOI:** 10.1155/2016/9345970

**Published:** 2015-12-20

**Authors:** Hiroshi Nagahisa, Kazuma Okabe, Yoshihito Iuchi, Junichi Fujii, Hirofumi Miyata

**Affiliations:** ^1^Biological Sciences, Graduate School of Medicine, Yamaguchi University, Yamaguchi, Yamaguchi Perfecture 753-8515, Japan; ^2^Biological Chemistry, Faculty of Agriculture, Yamaguchi University, Yamaguchi, Yamaguchi Perfecture 753-8515, Japan; ^3^Department of Biochemistry and Molecular Biology, Graduate School of Medical Science, Yamagata University, Yamagata, Yamagata Perfecture 990-9585, Japan

## Abstract

Cu/Zn superoxide dismutase (SOD1) knockout (KO) mice are known as an aging model in some aspects, but the damage and regeneration process of each fiber type have not been sufficiently studied. In this study, we investigated the damage and satellite cell state of the gastrocnemius muscle in SOD1 KO mice (6 months old) using immunohistochemical staining and real-time RT-PCR. The proportion of central nuclei-containing Type IIx/b fibers in the deep and superficial portions of the gastrocnemius muscle was significantly higher in SOD1 KO than control mice. The number of satellite cells per muscle fiber decreased in all muscle fiber types in the deep portion of the gastrocnemius muscle in SOD1 KO mice. In addition, the mRNA expression levels of Pax7 and myogenin, which are expressed in satellite cells in the activation, proliferation, and differentiation states, significantly increased in the gastrocnemius muscle of SOD1 KO mice. Furthermore, mRNA of myosin heavy chain-embryonic, which is expressed in the early phase of muscle regeneration, significantly increased in SOD1 KO mice. It was suggested that muscle is damaged by reactive oxygen species produced in the mitochondrial intermembrane space in Type IIxb fibers, accelerating the proliferation and differentiation of satellite cells through growth factors in SOD1 KO mice.

## 1. Introduction

Reactive oxygen species (ROS) are associated with cardiovascular and neurodegenerative diseases developing with aging [1]. ROS also inhibit the normal skeletal muscle regeneration process, being involved in age-related skeletal muscle weakness, that is, sarcopenia [2]. Generally, when skeletal muscle is damaged by stimulation, such as an overload, satellite cells, which are dormant myogenic stem cells, are activated. Activated satellite cells supply myonuclei to damaged muscle fibers through proliferation and differentiation, contributing to skeletal muscle repair. Some proliferated satellite cells do not differentiate into myonuclei, and they return to the dormant state and maintain a satellite cell pool for regeneration as a self-renewal system. Since ROS production markedly increases with aging [3], interference with the regeneration process described above by ROS may be a cause of skeletal muscle weakness.

Many tissues including skeletal muscle possess a superior system to control ROS, and superoxide dismutase (SOD) is one of the enzymes playing a control role. SOD mediates the disproportional reaction of converting oxygen, a species of ROS, to hydrogen peroxide in the body. There are isoforms of SOD: Cu-Zn SOD (SOD1) localized in the cytoplasm and mitochondrial intermembrane space and Mn SOD (SOD2) localized in mitochondria. Previous studies reported that SOD1 deficiency accelerated aging-related muscle weight reduction and the accumulation of oxidative damage in mice [4], suggesting that these were caused by superoxide increased by SOD1 deficiency and its secondary product. Thus, SOD1 KO mice are a useful model to investigate the influence of ROS and aging on the regeneration process of skeletal muscle. However, the muscle fiber regeneration process with satellite cell dynamics in SOD1 KO mice has not been sufficiently studied. In this study, SOD1 deficiency-induced structural and functional changes in muscle cells were investigated using immunohistochemical techniques and real-time RT-PCR.

## 2. Materials and Methods

All procedures were approved by the Animal Welfare and Ethics Committee of the Yamaguchi University and followed the American Physiological Society's Animal Care Guidelines.

### 2.1. Animals and Muscle Sampling

Seven* SOD1*−/− (SOD1 KO) mice [5] and seven control (CTL; C57BL/6 strain) mice were used in this study (males, 6.2 ± 0.2 months old).* SOD1*+/− b129Sv mice purchased through Jackson Laboratories (Bar Harbor, ME, USA) were backcrossed more than 8 times with C57BL/6 males and bred at our institute. All mice were maintained in a room controlled temperature and 12 h : 12 h light/dark cycle and unrestricted access to food and water. All animals were anaesthetized with pentobarbital sodium (60 mg/kg, intraperitoneally), and then left and right gastrocnemius muscles were removed. All muscle samples were frozen by liquid nitrogen and stored at −80°C until analyzed.

### 2.2. Immunohistochemical Analysis

**Figure 1:**
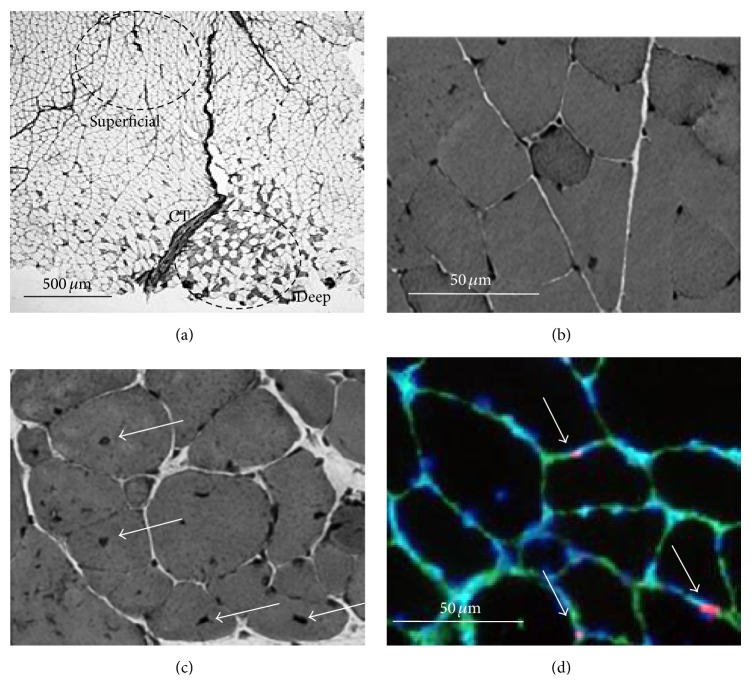
Histochemical staining sections of gastrocnemius muscle in mice. (a) Antimyosin heavy chain IIa; (b) and (c) hematoxylin and eosin staining; (d) identification of satellite cells by immunohistochemical triple staining. Only Type IIx/b fibers were present in the muscle (most superficial portion in (a)), while muscle fibers in the deep portion consisted of Types I, IIa, and IIx/b (small portion deeper than connective tissue (CT)). Many fibers with central nuclei (white arrows) were found in Cu/Zn superoxide dismutase (SOD1) knockout (KO) mice (c) but not in control (CTL) mice (b). Satellite cells (white arrows) were identified as stained positive for both DAPI (blue) and Pax7 (red) at the periphery of each fiber beneath the basal lamina (green) in (d).

Serial 10 *μ*m cross sections of the right muscle were obtained on a cryostat (CM510; Leica, Wetzlar, Germany) at −20°C. The sections were warmed to room temperature (RT) and then preincubated in 1% normal goat serum (EMD Millipore, Billerica, MA) in 0.1 M phosphate buffered saline (PBS; pH 7.6) at RT for 10 min. The primary monoclonal antibody was then applied: either (1) fast myosin (1 : 2000; Sigma, St. Louis, MO), which specifically reacts with the myosin heavy chain- (MHC-) IIa and IIx, or (2) SC-71 (1 : 1000; Developmental Studies Hybridoma Bank, Iowa City, IA), which specifically reacts with MHC-IIa. The sections were incubated in these primary antibodies overnight at RT and incubated with a secondary antibody (goat anti-mouse IgG) conjugated with horseradish peroxidase (HRP, Bio-Rad, Hercules, CA, 1 : 1,000) at RT for 3 hours. Diaminobenzidine tetrahydrochloride was used as a chromogen to localize HRP. Images of the stained muscle fibers were recorded with a photomicroscopic (E600; Nikon, Tokyo, Japan) image processing system (DS-U1; Nikon). The fibers were classified as Type I, IIa, or IIx/b fibers based on their immunohistochemical staining properties, and population and cross-sectional areas (CSAs) of each muscle fiber type were calculated in deep and superficial portions (Figure 1(a)).

### 2.3. Muscle Nuclei and Satellite Cell Identification

In another serial section, hematoxylin and eosin (HE) staining was conducted based on standard procedures. Number of nuclei and percentage of fibers with central nuclei were calculated for the 3 fiber types separately (Figures 1(b) and 1(c)).

Another serial section was fixed in 4% paraformaldehyde in 0.1 M PBS at RT for 10 min. These sections were preincubated in blocking solution containing 10% normal goat serum (EMD Millipore) and 2% bovine serum albumin (BSA; Sigma) in PBS at RT for 30 min. Each section was incubated for 1 hour at RT in the primary antibodies, a mouse anti-Pax7 (1 : 1000; Developmental Studies Hybridoma Bank) and a rabbit anti-laminin (1 : 1000; Sigma) diluted in 2% bovine serum albumin/PBS. The sections were incubated in appropriate secondary antibodies: Cy3-conjugated AffiniPure goat anti-mouse IgG (1 : 1000; Jackson ImmunoResearch, West Grove, PA) for Pax7 and AlexaFluor488 goat anti-rabbit IgG (1 : 1000; Molecular Probes, Eugene, OR) for laminin, respectively. After incubation, the sections were stained with 4,6-diamidino-2-phenylindole (DAPI, Molecular Probes) diluted in PBS at RT for 5 min. Images for anti-Pax7, antilaminin, and DAPI were merged with an image processing software (Adobe Photoshop software CS2) and used for quantification of satellite cells. Satellite cells were identified as stained positive for both DAPI and Pax7 at the periphery of each fiber beneath the basal lamina. The numbers of satellite cells/fiber were calculated for the 3 fiber types separately (Figure 1(d)).

### 2.4. RNA Isolation and Real-Time RT-PCR

Total RNA was extracted from the left muscles with TRIZOL reagent (Invitrogen, Carlsbad, CA). The purity and quantity of total RNA were determined by measuring the absorbance of aliquots at 260 and 280 nm. Total RNA was then treated for 30 min at 37°C with TURBO DNase (Ambion-Life Technologies, Austin, TX) to remove genomic DNA from samples. DNase-treated RNA (0.5 *μ*g) was used to synthesize first-strand cDNA with an Exscript RT reagent Kit (TaKaRa Bio, Otsu, Japan). Thereafter, the cDNA products were analyzed by real-time PCR using the SYBR Green PCR Master Mix protocol in a StepOne Real-Time PCR System (Applied Biosystems Japan, Japan).

The amplification program included an initial denaturation step at 95°C for 10 min, 40 cycles of denaturation at 95°C for 30 sec, and annealing/extension at 58°C for 1 min. The amount of glyceraldehyde-3-phosphate dehydrogenase (GAPDH) mRNA was estimated as an internal control. Each mRNA was normalized to GAPDH by subtracting the cycle threshold (Ct) value of GAPDH from the Ct value of the gene target [ΔCt (target)]. The relative expression of the target gene was calculated as the relative quantification (RQ) value for CTL value. Following the relative expression, dissociation-curve analysis detected no nonspecific amplification in cDNA samples.

The sequences of the specific primers used in this study were presented in Table 1. Each PCR primer was designed by Primer Express software (v3.0; Applied Biosystems), and the oligonucleotides were purchased from FASMAC (Kanagawa, Japan).

### 2.5. Statistics

All data are presented as the mean ± SE. Data obtained from the histochemical analysis were analyzed with one-way ANOVA followed by *t*-test with Bonferroni adjustment. A Wilcoxon's signed-rank test was used to compare differences in mRNA expressions between SOD1 KO and CTL mouse. Statistical significance was set at *P* < 0.05.

## 3. Results

### 3.1. Body and Muscle Weights

Although the difference was not significant, the body weight was 17% lower in SOD1 KO than CTL mice (CTL: 33.2 ± 2.4 g, SOD: 27.4 ± 2.0%). The muscle weight also tended to decrease (CTL: 62.8 ± 4.9 mg, SOD: 55.6 ± 3.3 mg), and the relative muscle weight for the body weight was about 0.2% in both groups.

### 3.2. Muscle Fiber Type Population and Area

Based on the results of immunohistochemical staining, the gastrocnemius muscle was divided into the deepest and superficial portions, and 300 fibers were analyzed in each portion, setting the baseline to the parameters in connective tissue in the deep portion.

Regarding the muscle fiber type population, Type IIa fibers increased and Type IIx/b fibers decreased in the deep portion of the gastrocnemius muscle in SOD1 KO mice compared to those in CTL mice. No change was noted in Type I fiber composition. Only Type IIx/b fibers were present in the superficial portion of the gastrocnemius muscle in both mouse groups.

**Figure 2:**
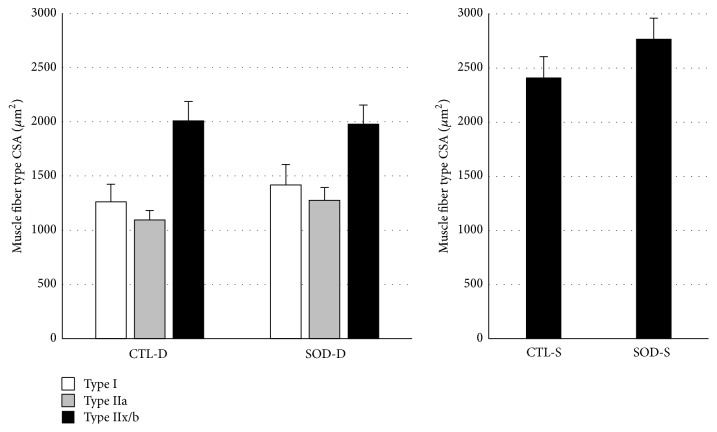
Comparison of cross-sectional area (CSA) in each fiber type between CTL and SOD1 KO mice. No significant differences were noted in the CSA of all fiber types in both portions. D: deep portion; S: superficial portion. Values are mean ± SE.

As shown in Figure 2, the CSA in the deep portion of the gastrocnemius muscle tended to increase in SOD1 KO mice compared to those in CTL mice, but no significant difference was noted in any muscle fiber type. Similarly, no significant change was noted in Type IIx/b CSA in the superficial portion.

### 3.3. Myonuclei and Satellite Cells

**Figure 3:**
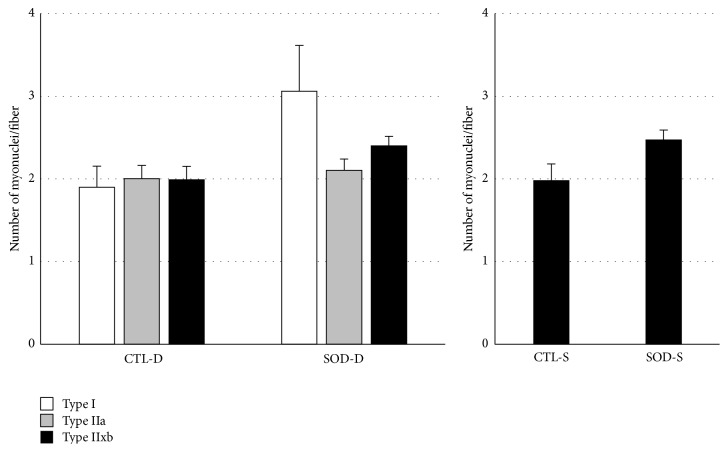
Comparison of number of myonuclei in each fiber type between CTL and SOD1 KO mice. Myonuclear number of all fiber types tended to increase in both portions in SOD1 KO mice compared to those in CTL mice. D: deep portion; S: superficial portion. Values are mean ± SE.

As shown in Figure 3, the tendency of the myonuclear number was similar to that of the CSA: the myonuclear number increased in all fiber types in the deep portion in SOD1 KO mice compared to those in CTL mice, but the increases were not significant. A similar tendency was noted in Type IIx/b fibers occupying the superficial portion.

**Figure 4:**
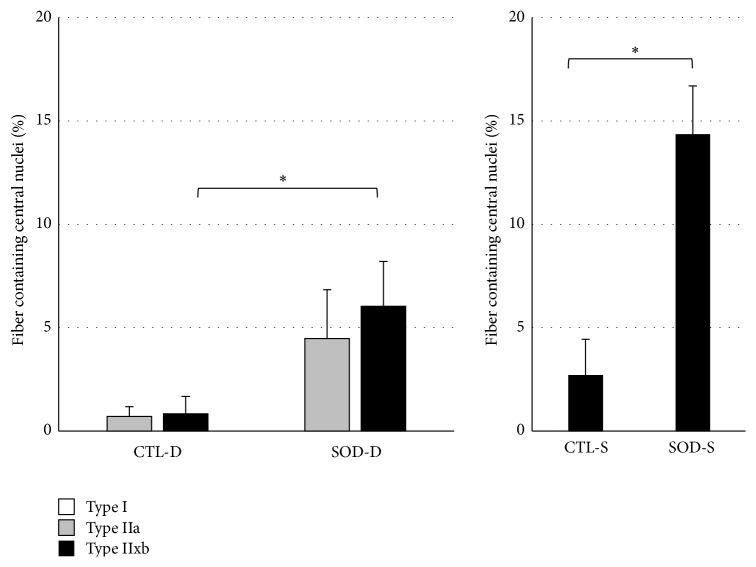
Comparison of proportion of central nuclei-containing muscle fibers in both portions between CTL and SOD1 KO mice. The proportions in Type IIx/b fibers were significantly higher in SOD1 KO than CTL mice. D: deep portion; S: superficial portion. Values are mean ± SE, ^*∗*^ *P* < 0.05.

As shown in Figure 4, the proportion of Type IIx/b fibers containing a central nuclei (%) in the deep portion was significantly higher in SOD1 KO than CTL mice. No central nuclei was present in Type I fibers in the deep portion in either group, but it tended to increase in Type IIa fibers in SOD1 KO mice compared to that in CTL mice. In addition, the proportion of central nuclei-containing Type IIx/b fibers in the superficial portion was significantly higher in SOD1 KO than CTL mice.

**Figure 5:**
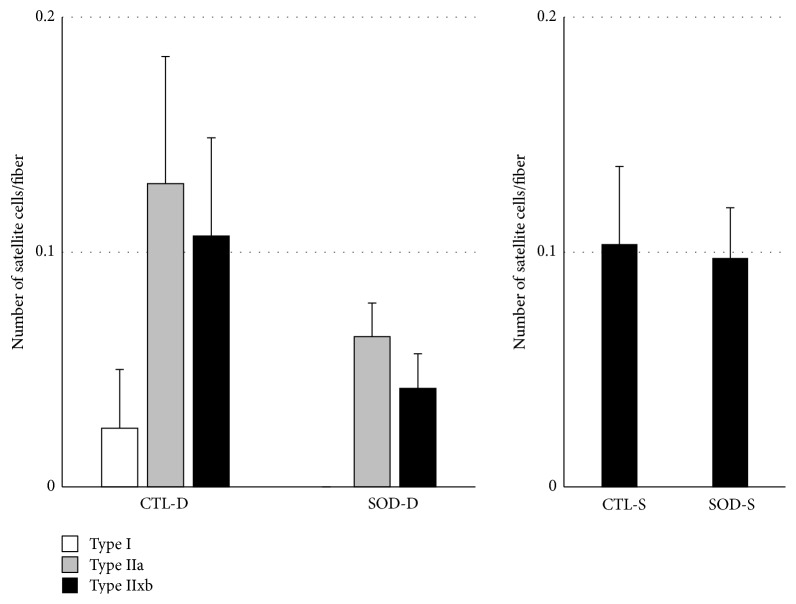
Comparison of number of satellite cells in both portions between CTL and SOD1 KO mice. Number of satellite cells per specified number of muscle fibers tended to decrease in SOD1 KO mice compared to that in CTL mice in all muscle fiber types. D: deep portion; S: superficial portion. Values are mean ± SE.

As shown in Figure 5, the number of satellite cells per specified number of muscle fibers tended to decrease in SOD1 KO mice compared to that in CTL mice in all muscle fiber types. A similar tendency was noted in Type IIx/b fibers in the superficial portion.

### 3.4. Satellite Cell-Related mRNA Expression

**Figure 6:**
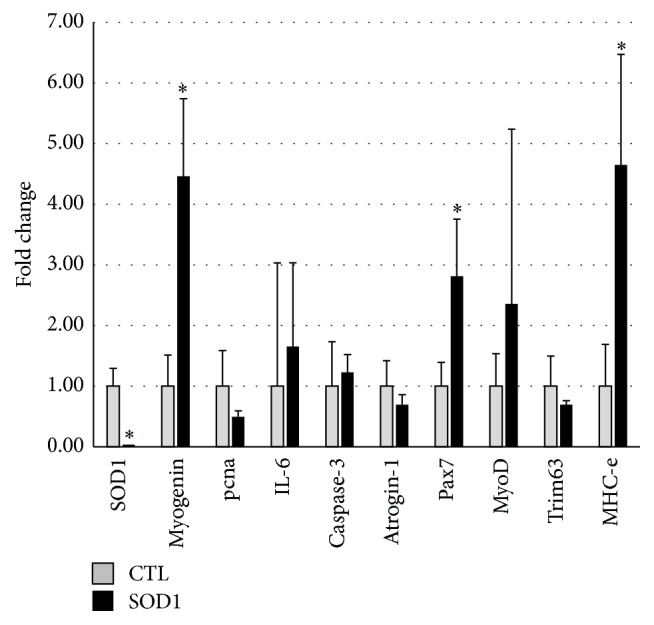
Comparison of mRNA expressions between CTL and SOD1 KO mice. Expressions of the target gene in SOD1 KO were presented as the relative values (fold change) for CTL mouse. Expressions of myogenin, Pax7, and MHC-e were significantly enhanced in SOD1 KO mice compared to those in CTL mice. Values are mean ± SE. ^*∗*^ *P* < 0.05, versus CTL.

Since it is difficult to accurately divide the deep and superficial portions for real-time RT-PCR, the 2 regions were combined in this analysis, and the expression level was presented as a value relative to that in CTL mice (Figure 6). SOD1 mRNA SOD1 expression was not detected in SOD1 KO mice. Expressions of Pax7, which is expressed in the activation and proliferation states of satellite cells, and myogenin, which is expressed in the differentiation state, were significantly enhanced in SOD1 KO mice compared to those in CTL mice. Similarly, the expression level of MyoD, which is expressed in the proliferation and differentiation states, tended to increase. The expression level of an inflammatory cytokine, IL-6, which is a satellite cell activator, tended to increase in SOD1 KO mice. The expression level of MHC-embryonic (MHC-e), which is considered to be expressed in the early phase of muscle regeneration, significantly increased in SOD1 KO mice.

## 4. Discussion

### 4.1. SOD1 Deficiency-Associated Muscle Fiber Damage

The rate of central nuclei-containing Type II fibers in the superficial and deep portion of the gastrocnemius muscle was higher in SOD1 KO than CTL mice, and the rate of central nuclei-containing Type IIx/b fibers was significantly higher, being consistent with the results of previous studies in which central nucleus increased in the flexor digitorum brevis muscle in SOD1 KO mice [6]. Generally, central nuclei are observed in muscle fibers in the regeneration process. Therefore, the high proportion of central nuclei-containing fibers in SOD1 KO mice may have been due to marked oxidative damage by ROS compared to that in CTL mice. High H_2_O_2_ production in Type II fibers, particularly Type IIb fibers, was demonstrated in a preceding study [7], in which it was also suggested that superoxide production occurs in the intermembrane space in Type IIb fibers, not in the matrix, being different from that in other fiber types. These findings suggest that the necessity of SOD1 present in the mitochondrial intermembrane space is higher for Type IIb than other fiber types. However, high ROS production in Type IIb fibers contradicts the fact that Type I fibers contain more mitochondria, the main source of ROS production. This contradiction may be related to differences in the partial pressure of oxygen among muscle fiber types and ROS production under a hypoxic condition. The partial pressure of oxygen in the microvasculature is low in the gastrocnemius muscle, rich in Type II fibers, compared to the soleus muscle and diaphragm, rich in Type I fibers, and a lower partial pressure of oxygen in a region containing many fast muscle fibers than that in a region containing less fast muscle fibers within the gastrocnemius muscle has been reported [8]. It is also known that ROS production is inhibited by lowering the electron current passing through the electron transport chain to adapt to a hypoxic condition of the tissue. Under a hypoxic condition, the mitochondrial electron transport efficiency decreases, and ROS are produced through passing electrons from complexes I and III to molecules different from normal receiver molecules [9]. In addition, an increase in ROS by a complex III inhibitor, antimycin, and the prevention of oxidative stress-associated ischemia-reperfusion injury by decreasing metabolic dependence on the electron transport chain have been clarified [10, 11]. Furthermore, it has been shown that the ability of oxidative fibers to upregulate NO-induced antioxidative enzymes is higher than that of glycolytic fibers [12]. Considering these comprehensively, the differences in the number of central nucleus among the fiber types in SOD1 KO mice may have been due to hypoxia-induced or low antioxidative enzyme level-associated ROS production in the mitochondrial intermembrane space in Type II fibers, particularly Type IIx/b fibers.

### 4.2. Influence of SOD1 Deficiency on Satellite Cells

On analysis of mRNA expression using real-time RT-PCR, significant increases in the Pax7, myogenin, and MHC-e mRNA levels and a slight increase in the MyoD mRNA level were observed in the gastrocnemius muscle of SOD1 KO mice. Pax7, MyoD, and myogenin are markers detected in the resting/proliferation, proliferation/differentiation, and differentiation states of satellite cells, respectively. MHC-e is expressed in the fetal period and muscle regeneration before differentiation into adult fiber types. Since these factors were upregulated, satellite cells may have been activated in SOD1 KO mice compared to those in CTL mice, and the muscle regeneration process may have been enhanced. Actually, the myonuclear number per muscle fiber tended to increase in SOD1 KO mice, which may have resulted from the differentiation of satellite cells into myonuclei.

There are many common points between SOD1 deficiency and aging-related changes, such as increases in ROS production and inflammatory cytokines and reduction of the muscle function [2]. A study on satellite cell activation and the stem cell niche clarified that MyoD and myogenin mRNA expressions in satellite cells are more markedly enhanced in senescence compared to those in early life, showing that the activation and differentiation of satellite cells are promoted in senescence, whereas the Pax7-positive rate of satellite cells decreased in senescence, suggesting that the replication competence of satellite cells decreases with aging, reducing the number of satellite cells [13]. In addition, stem cell niche-derived fibroblast growth factor-2 (FGF2) was identified as the main cause of these aging-related changes in the study. In our study, the number of satellite cells per muscle fiber decreased in SOD1 KO mice compared to that in the CTL mice. It has also been suggested that an increase in ROS production leads to an increase in FGF2 production in sheep pulmonary arterial smooth muscle cells [14]. An increase in the IL-6 mRNA expression level was observed in our study. IL-6 is a satellite cell activator through the JAK/STAT3 signaling pathway (Janus kinase/signal transducer and activators of transcription 3) [15, 16], and it has been demonstrated to be a regulatory factor essential for skeletal muscle hypertrophy in a compensatory overload experiment using IL-6 KO mice [17].

Therefore, enhanced MyoD, myogenin, and MHC-e mRNA expressions in SOD1 KO mice observed in our study may have been due to the activation of satellite cells by constantly increased FGF2 and IL-6 induced by constantly increased ROS.

### 4.3. Specificity of SOD KO Mouse Model

SOD1 KO mice are considered a useful model to investigate the association between an increase in ROS production and muscular atrophy, but responses specific to this model are also observed. For example, increases in the PGC1*α* mRNA expression level and mitochondria in SOD1 KO mice have been reported [18], whereas reduction of the PGC1*α* expression level and mitochondrial function by increased ROS production in response to aging-related changes, disuse, and hypoxic stimulation has been observed [18–23]. Moreover, slowed muscle contraction-inducing adaptive responses [24] and the accumulation of mitochondria below the muscle cell membrane have been observed in SOD1 KO mice [18, 25]. It has been suggested that constant ROS production interferes with ROS production during muscle contraction, slowing muscle contraction-inducing adaptive responses [24]. Actually, another study [6] reported the absence of an increase in ROS after contraction in SOD1 KO mice. It has also been clarified that the muscle contraction-inducing ROS production in CTL mice is higher than that in resting SOD1 KO mice [6], and expression of proteins with antioxidative actions, such as SOD2 and heat shock proteins, is enhanced in SOD1 KO mice compared to those in control mice [24]. These phenomena indicate that constant ROS production markedly changes the antioxidative defense system, to which attention should be paid when SOD1 KO mice are considered as an aging model.

## 5. Conclusion

The proportion of central nuclei-containing Type IIx/b fibers in the deep and superficial portions of the gastrocnemius muscle was significantly higher in SOD1 KO than CTL mice. The mRNA expression levels of Pax7, myogenin, and MHC-embryonic significantly increased in SOD1 KO mice. It was suggested that muscle is damaged by ROS produced in Type IIxb fibers, accelerating the proliferation and differentiation of satellite cells in SOD1 KO mice.

## Figures and Tables

**Table 1:** Real-time RT-PCR primer sequences.

GAPDH	F	CATGGCCTTCCGTGTTCCTA
	R	GCGGCACGTCAGATCCA

SOD1	F	GCCCGGCGGATGAAG
	R	CCTTTCCAGCAGTCACATTGC

Myogenin	F	AGCATCACGGTGGAGGATATG
	R	CAGTTGGGCATGGTTTCGT

IL-6	F	CCACGGCCTTCCCTACTTC
	R	TTGGGAGTGGTATCCTCTGTGA

Pax7	F	AAAAAACCCTTTCCCTTCCTACA
	R	AGCATGGGTAGATGGCACACT

MyoD	F	GCCGGTGTGCATTCCAA
	R	CACTCCGGAACCCCAACAG

MHC-e	F	GAGCAGCTGGCGCTGAA
	R	TCTGATCCGTGTCTCCAGTTTCT

SOD1: superoxide dismutase 1; IL-6: interleukin-6; Pax7: paired box 7 protein; MyoD: myogenic determination; MHC-e: myosin heavy chain-embryonic.
